# Enhanced light focusing inside scattering media with shaped ultrasound

**DOI:** 10.1038/s41598-023-38598-5

**Published:** 2023-07-17

**Authors:** Blanca Mestre-Torà, Martí Duocastella

**Affiliations:** 1 Department of Applied Physics, Universitat de Barcelona, C/Martí i Franquès 1, 08028 Barcelona, Spain; 2 Institut de Nanociència i Nanotecnologia (In2UB), Universitat de Barcelona, 08028 Barcelona, Spain

**Keywords:** Applied optics, Imaging and sensing

## Abstract

Light focusing is the primary enabler of various scientific and industrial processes including laser materials processing and microscopy. However, the scattering of light limits the depth at which current methods can operate inside heterogeneous media such as biological tissue, liquid emulsions, and composite materials. Several approaches have been developed to address this issue, but they typically come at the cost of losing spatial or temporal resolution, or increased invasiveness. Here, we show that ultrasound waves featuring a Bessel-like profile can locally modulate the optical properties of a turbid medium to facilitate light guiding. Supported by wave optics and Monte Carlo simulations, we demonstrate how ultrasound enhances light focusing a factor of 7 compared to conventional methods based on placing optical elements outside the complex medium. Combined with point-by-point scanning, images of samples immersed in turbid media with an optical density up to 15, similar to that of weakly scattering biological tissue, can be reconstructed. The quasi-instantaneous generation of the shaped-ultrasound waves, together with the possibility to use transmission and reflection architectures, can pave the way for the real-time control of light inside living tissue.

The focusing of light down to a micrometric region is of prime importance in several scientific and industrial processes. It enables capturing the morphology and dynamics of cells with optical microscopy^1^ or spectroscopy techniques^2^, the fabrication of parts using three-dimensional printers^3^, or the treatment of skin via laser irradiation^4^. Unfortunately, many relevant materials ranging from biological tissue to liquid emulsions are complex media, namely heterogeneous systems with locally diverse optical properties. As a result, when light propagates inside such media, it is rapidly scattered well before optical absorption dominates. Specifically, the number of ballistic photons—photons not deviated—decreases exponentially, with photons underlying one scattering event after an average distance called the mean free path (MFP). The specific value of the MFP depends on the wavelength of light and material properties. For instance, the MFP of biological tissue when operating in the so-called biological windows—infra-red wavelengths, when light can penetrate the most^5^—is 0.16 mm for skin and only 0.06 mm for lungs^6^. Similarly, the MFP of milk is about 0.33 mm^7^. Therefore, current light-based methods are only optimized to operate at depths below a fraction of a millimeter inside complex media^8^. This represents a strong limitation toward the use of optical techniques as fast, non-invasive, and precise characterization and fabrication tools.

Several strategies have been developed to overcome the challenge of focusing light inside scattering media. An example includes endoscopy methods, based on using fiber bundles called endoscopes that are directly inserted into the complex media for guiding the light. While they provide an unlimited penetration depth, they are intrinsically invasive, and thus unsuitable to study delicate systems such as the brain, organoids, or small multicellular constructs in general^8^. An alternative technique to focus light inside tissue is optical wavefront shaping^9,10^. Its underlying principle is to consider scattering a deterministic process that can be undone. By collecting information from the complex medium (recording step) and using it to locally change the phase of a laser beam (playback step), this technique can achieve impressive results, with focus confinement of some microns well beyond several MFP^11,12^. Unfortunately, retrieving the “scattering fingerprint” of the tissue normally requires a guide-star, that is, the emission of light from a point inside the medium^13^. Obtaining a non-invasive, highly confined guide-star has proven challenging; recent successful strategies, including iterative methods using ultrasonically modulated light, are time-consuming and, consequently, cannot cope with the rapid dynamics of living systems or turbid media^14,15^. Similarly, using non-diffracting and self-healing beams can help to partially compensate for scattering, but they come at the expense of losing axial resolution or increased complexity of the focusing element^16^. Simply put, deep light focusing with existing methods is achieved by sacrificing core advantages of optical methods such as losing spatial or temporal resolution, or by becoming invasive.

A recent attempt to balance the tradeoff between invasiveness, focusing depth, and spatiotemporal resolution is using ultrasound to modulate the optical properties of the complex medium itself^17,18^. In this way, the induced refractive index modulation effectively acts as an embedded waveguide or lens inside the medium, helping to compensate for scattering^19^. Although this phenomenon has been qualitatively studied for transmitted light, the focusing performance of such ultrasonically guided light in more realistic scenarios—where transmitted light is not accessible—remains largely unexplored. In this work, we fill this void and present a comprehensive characterization of the focusing capabilities of ultrasound for transmitted and reflected light in different scattering media. We compare our experiments with conventional focusing methods based on external optical elements and demonstrate up to a factor of 7 improvement in light confinement. Our results are in good agreement with wave optics and Monte Carlo simulations, which help to provide the physical foundation for the observed light-confinement effect. Combined with a point-by-point scanning system, we show how ultrasound focusing enables visualizing samples inside turbid media at a micrometric resolution that would remain hidden with conventional imaging methods.

## Results

### Light focusing inside an ultrasound-modulated medium

**Figure 1:**
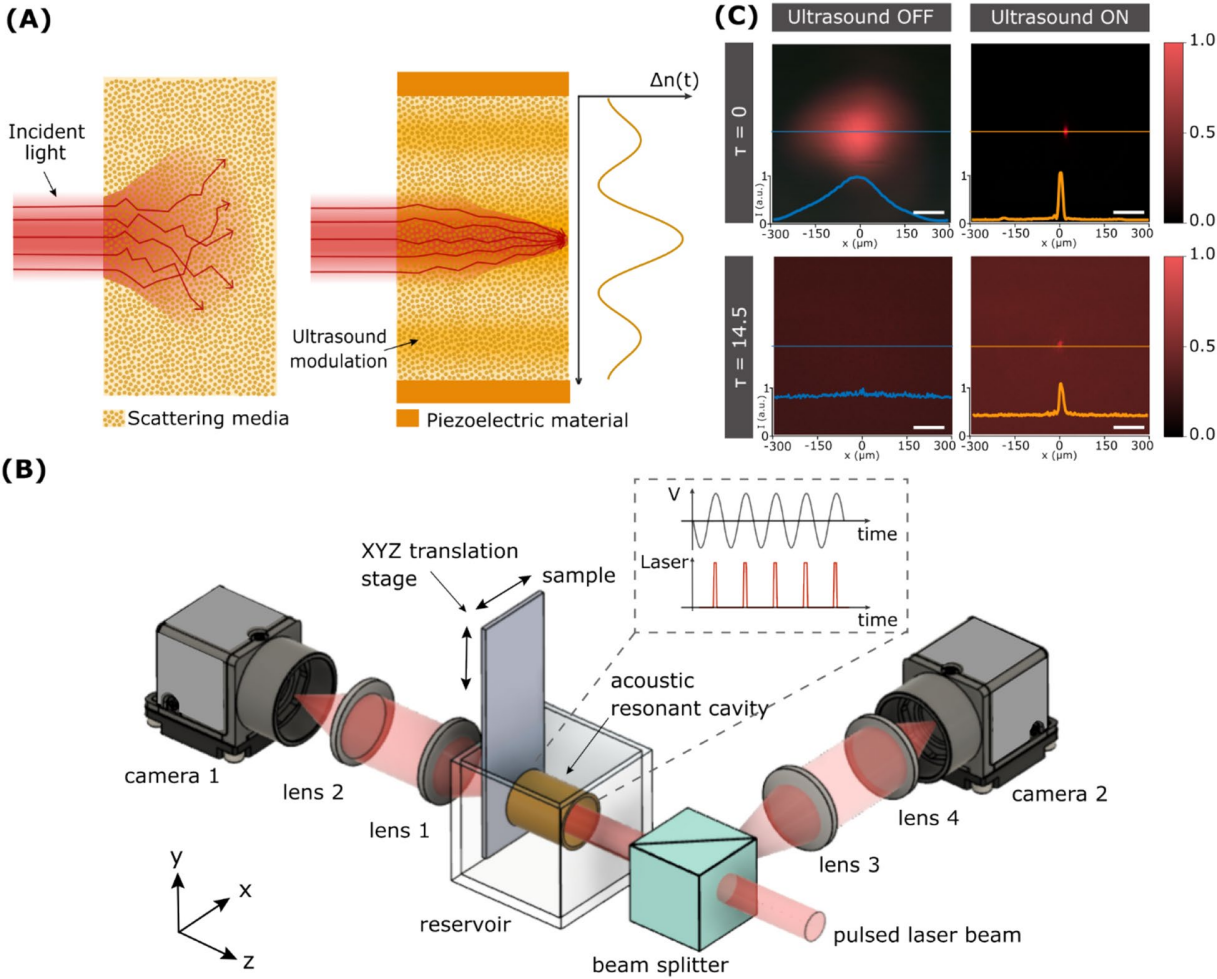
Focusing of light with ultrasound. (**A**) Schematic representation of the scattering process that light undergoes when propagating inside a heterogeneous medium (left), and the waveguiding effect that ultrasound produces (right). (**B**) Scheme of the experimental setup used. The central element is the piezoelectric cylinder filled with a fluid that, when driven on resonance, generates ultrasound waves in its interior. (**C**) Experimental optical micrographs of a pulsed laser beam at the output of the cavity with and without ultrasound inside a homogeneous medium (water) and a turbid medium (milk/water mixture). Ultrasound enables light confinement to a spot of about 25 µm even in the presence of scattering. Scale bars are 100 μm.

The core concept of light focusing with ultrasound is to use pressure waves with a selected intensity profile to induce local variations in the density of a medium, and consequently, its refractive index^20^. The interaction of light with such a graded refractive index medium can be used to split, focus or guide a laser beam via the acousto-optic effect^20^. These phenomena are usually observed inside homogeneous media. When applying ultrasound modulation in a scattering medium, we hypothesize that proper selection of the graded refractive index will help to compensate for the deviation of scattered photons. In other words, the competition between light scattering and ultrasound-based light guiding will help to enhance the number of photons that reach the desired focal spot, as shown in Fig. 1A.

To generate the refractive index distribution inside the complex medium, we used a piezoelectric cylindrical cavity. When filled with a liquid and driven on resonance (4 MHz), radial standing ultrasonic waves are obtained in the cavity^21^, which induce a periodic change in the liquid refractive index given by:^22^

1 $$n\left( {r,t} \right) = n_{0} + n_{A} \cdot J_{0} \left( {kr} \right)\cdot\cos \left( {\omega t} \right),$$

where ${n}_{0}$ corresponds to the static refractive index of the liquid, ${n}_{A}$ the amplitude of the refractive index change that depends on the driving amplitude voltage, ${J}_{0}$ the Bessel function of first kind, $k$ is the acoustic wave vector, and $\omega$ the driving frequency. This refractive index acts as a time-dependent gradient index of refraction (GRIN) lens^23,24^. By using pulsed illumination shorter than the cavity oscillation time, the interaction of light with the instantaneous refractive index described in Eq. 1 can result in a Bessel-like beam focused along the optical axis^23^. The optical properties of the so-generated focused beam depend on the driving frequency as well as the amplitude driving voltage—the latter ultimately determines ${n}_{A}$.

A scheme of the optical system to characterize the focusing of light with ultrasound is shown in Fig. 1B. The central element consists of the piezoelectric cylindrical cavity placed in a transparent chamber that can be filled with the desired medium (see Supplementary Fig. S1). In current experiments, we used a water/milk mixture whose scattering properties could be tuned by adjusting the concentration of milk (see Materials and Methods). Note that such a solution constitutes a turbid medium, with multiple scattering centers constantly moving. Thus, wavefront shaping or other intrinsically slow techniques for deep light focusing could not be used in this case. An XYZ translation stage allows micrometric control of the sample, placed just after the cavity. Importantly, the system features two detection arms, each with its camera, for operation in both transmission and reflection modes. Figure 1C shows an example of the light-focusing effects with ultrasound. As expected, in the absence of ultrasound, a collimated laser beam passing through a water-filled cavity (homogeneous medium) experiences no significant distortion. Thus, the beam measured after the cavity preserves its initial full width at half maximum (FWHM) of 300 µm. Instead, by turning the ultrasound ON, the laser beam can be focused down to a spot of 25 µm at the output of the cavity. This is a well-known effect that forms the basis of the varifocal lens named TAG (Tunable Acoustic Gradient) lens^25^, which uses the acousto-optic effect for light focusing at microsecond time scales.

Repeating the experiment with the cavity filled with an inhomogeneous medium leads to significantly different results (Fig. 1C, bottom). In this case, the water/milk mixture had an optical thickness (τ) of 14.5. Note that we used τ as the adimensional parameter to quantify scattering, defined as ${\mu }_{s}d$, where d is the thickness of the medium and ${\mu }_{s}$ its scattering coefficient measured by the attenuation of light intensity in the medium (see Supplementary Information and Fig. S2). The optical thickness can also be regarded as the number of scattering events experienced by light inside a medium. When ultrasound is OFF, the light attenuation caused by scattering produces a decrease in light intensity, together with a significant spread of the beam spot. Such a deterioration in light confinement proves the negative effects that scattering can have in light focusing and imaging applications. Remarkably, when the ultrasound is ON, the focused beam at the output of the cavity can still be discerned from the background and its diameter is maintained close to 25 µm, albeit with an increment of the background photons. These results are in line with those recently reported in literature^17–19^. Still, there are three main critical aspects that have yet to be addressed. First, an appropriate model to explain ultrasound-light focusing. Secondly, a comparison of ultrasound focusing with traditional methods based on external optical elements. Thirdly, a demonstration of the possibility to work with the detector placed on the same side where light is incident – critical given the difficulty to access transmitted information in most realistic scenarios.

### Simulation of ultrasound-based light focusing

**Figure 2:**
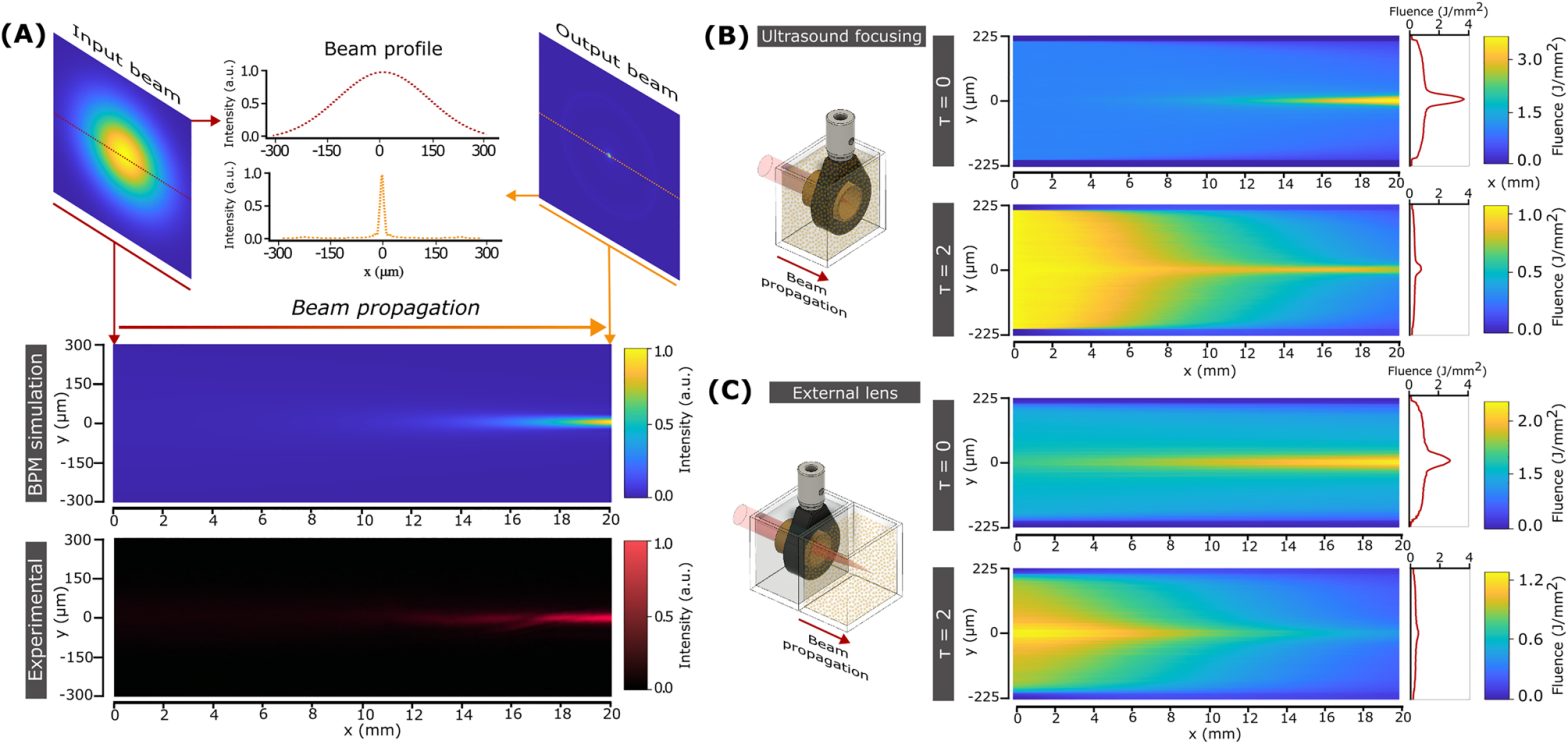
Simulations of the ultrasound focusing of light. (**A**) Beam Propagation Method simulations of the focusing of a laser beam inside a homogeneous medium modulated by ultrasound (top). An initial 300 µm beam can be confined to a spot of 25 µm. Experimental image of the light intensity profile inside the resonant cavity (bottom). (**B**) Monte Carlo simulations of ultrasound focusing inside a homogeneous (up) and scattering (down) media. (**C**) Monte Carlo simulations of traditional light focusing using an external optical element inside a homogeneous (up) and scattering media (down). Note that we used the same ultrasound cavity filled with water as the external lens. In all Monte Carlo simulations, the fluence of the beam at the input of the scattering media was selected to be 1 J/cm^2^. The insets on the right correspond to the light intensity profiles at the output of the cavity.

To model the effects of ultrasound on the propagation of light, we used two different methods. The first one, called the Beam Propagation Method (BPM), is based on wave optics under the paraxial approximation and it is suitable for simulating non-scattering media. The simulation results for a water-filled cavity considering a modulation in refractive index given by Eq. 1 with ${n}_{A}$= 8·10^–5^, a driving frequency of 4 MHz (acoustic wavevector of 15.8 mm^-1^) and propagation distance of 2 cm (the length of the resonant cavity) are shown in Fig. 2A. Notably, an input collimated beam with an FWHM of 300 µm is focused down to a well-defined spot of only 25 µm at the output. As expected in a GRIN-type medium, the confinement of light progressively increases during propagation. To verify the simulation results, we conducted an experimental measurement of the intensity of light within the cavity. Specifically, we captured a sequence of images at various positions inside the cavity using an external microscope (see Materials and Methods). The reconstructed intensity profile, shown in Fig. 2A-bottom, is in perfect agreement with the simulation.

While the behavior of light in an ultrasound-modulated medium can be properly simulated with BPM, the method cannot be applied to scattering media. For this challenging case, we employed Monte Carlo simulations based on the photon packet method^26^. The simulation geometry consisted of a triangular mesh where the refractive index given by Eq. 1 and scattering coefficient were locally specified (see Materials and Methods). Figure 2B-top shows the simulated behavior of light propagation in the medium with negligible scattering. The results are comparable with those obtained with the experiment and the BPM simulation, that is, the light is progressively focused as it propagates through the 2 cm modulated medium, down to a spot of about 35 µm. This validates the use of the Monte Carlo method to simulate photon transport driven by ultrasound. Next, we repeated the simulation in a scattering medium with an optical thickness of τ = 2 (Fig. 2B-bottom). In this case, and despite the attenuation caused by scattering, a considerable number of photons still reach the end of the cavity. In fact, it is still possible to discern the focused beam, which features the same size as in the non-scattering case but with a loss in focusing contrast (focused vs background photons) of 18% (see "Supplementary Information"). This is in striking difference with propagation without ultrasound, where almost no photons reach the end of the cavity (see Supplementary Fig. S3). These results support our initial hypothesis that an ultrasound-modulated medium effectively acts as an embedded waveguide, redirecting a fraction of the scattered photons to the area with the highest refractive index.

The question that remains to be answered is how advantageous ultrasound can be compared with traditional methods based on optical elements placed outside the scattering medium. To this end, we ran a Monte Carlo simulation where an external lens was used to focus light inside different complex media. We selected an ultrasound cavity filled with a homogeneous medium as the focusing lens. In this case, a Bessel-like beam is formed after the cavity, with a spot size of around 40 µm at 2 cm (see Fig. 2C-top). The Bessel-like beam propagation is in good agreement with experiment and BPM simulation (Supplementary Fig. S4). When simulating propagation in a scattering medium with τ = 2, the number of ballistic photons reduces, and at 2 cm the focusing contrast is reduced to 50% (see Fig. 2C-bottom). This significant loss of light is more than a factor of two higher than with ultrasound focusing. Notably, the benefits of ultrasound are still present for media with larger scattering, as shown in Supplementary Fig. S5 for a medium with τ = 10. At these conditions, while the external element fails to confine light, ultrasound can still obtain a well-defined spot at a depth of 2 cm.

### Optical characterization of the focusing system

**Figure 3:**
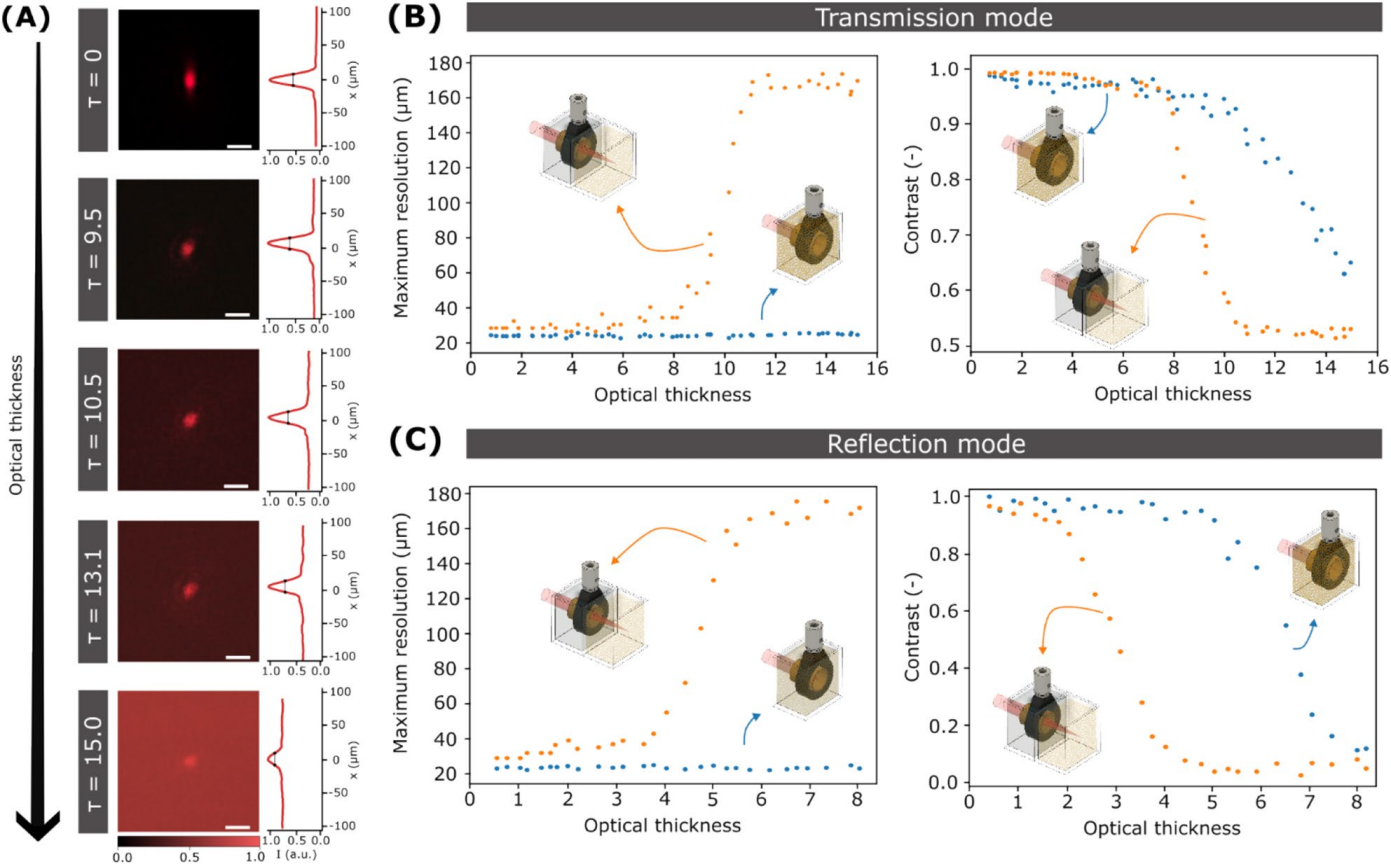
Optical performance of the ultrasound cavity for light focusing. (**A**) Optical images of the focused beam at the output of the cavity when filled with water/milk mixtures of different optical thicknesses. Scale bars are 40 µm. (**B**) Plots of the optical resolution (left) and contrast (right) of the system using ultrasound for focusing (blue bullet) and an external lens (orange bullet) as a function of the optical thickness of the medium when working in transmission mode and (**C**), in reflection mode.

An experimental evaluation of the optical performance of the ultrasound focusing system for different complex media is needed to further validate simulations. To this end, we measured the system response at a depth of 2 cm, just outside the cavity. Figure 3A shows the general effects of scattering on light confinement. As scattering increases, the focused spot size (point spread function (PSF) of the system) remains approximately constant with an FWHM of 25 µm, but the number of background photons increases. Such a loss in contrast will ultimately limit the focusing capability of the system—eventually, the ratio of focused vs background photons would be too low to discern light confinement. In current experiments, this limit was achieved for a scattering medium with an optical thickness (τ) above 15, in the range of weakly scattering tissue^27^.

Measuring the system's optical resolution can provide a more quantitative analysis of the scattering effects on ultrasound focusing. To this end, we determined the system modulation transfer function (MTF) and defined the cut-off frequency (the maximum spatial frequency the optical system can resolve, and the inverse of spatial resolution) at an MTF value of 0.1. We characterized the MTF with the slanted-edge method^28,29^. As its name indicates, this method is based on capturing images of a knife-edge target, which are processed following three steps (Supplementary Fig. S6). Here, we captured an image of a knife-edge target by scanning the sample across the focus of our system point-by-point (see Materials and Methods section)^28,29^. As shown in Fig. 3B, the spatial resolution of the ultrasound focusing system is around 25 µm and it remains constant when increasing the optical thickness of the medium, up to τ = 15.3. This behavior is in good agreement with the previous experiments and simulations.

To better contextualize these results, we compared them with the classical way of focusing light, that is, using an external optical element. For a fair comparison, we used the water-filled external cavity as described in the previous section. In this case, the system spatial resolution starts at around 25 µm, as with ultrasound focusing, but monotonically decreases with the optical thickness of the medium (Fig. 3B). More precisely, there is a steep transition from τ = 7 to τ = 12, where the system resolution rapidly decays from 35 to 170 µm. At this point and onwards, a diffusive regime is reached where noise dominates the measurement of the spatial resolution, which remains fixed at 170 µm. Such a deterioration in light confinement contrasts with the results of ultrasound focusing, where a gain in spatial resolution of up to a factor of 7 can be observed for a large optical thickness.

Even if the spot size obtained with ultrasound focusing does not seem to increase with optical thickness, there is a clear increase in the number of background photons (Fig. 3A). To quantify this phenomenon, we introduce a contrast parameter defined as the difference in light intensity between the focused photons (${I}_{max}$) and the background signal (${I}_{min}$) relative to the total intensity (${I}_{max}+{I}_{min}$)^30^:

2 $$Contrast = \frac{{I_{max} - I_{min} }}{{I_{max} + I_{min} }}$$

As expected, the contrast function extracted from the slanted-edge images decreases monotonically with the medium optical thickness (Fig. 3B). Still, there is a clear benefit of using ultrasound. With an external focusing element, the contrast decreases rapidly from nearly 1 to 0.5 as the optical thickness of the mixture varies from 8 to 10. Instead, during the same interval, ultrasound focusing only experiences a 20% loss in contrast.

All previous results involved light detection in a transmission configuration. However, in most applications the sample is only accessible from one side—this is typically the case in biological tissue constructs. To characterize the optical performance of our system in this configuration, we measured the MTF in reflection mode, with both illumination and detection placed on the same sample side. Because the beam is focused inside the scattering medium, interacts with the sample, and returns throughout the same path, light traverses the scattering medium twice. Figure 3C shows the resolution of the system working in reflection mode as a function of the optical thickness. The same trends as when working in transmission mode are observed. Thus, with ultrasound focusing the resolution of the system is maintained constant at a value of 25 µm. Instead, with an external focusing element resolution rapidly deteriorates, from around 40 µm to 170 µm once the optical thickness of the medium is larger than 4. Interestingly, measurements were only possible up to a value of the optical thickness of 8.2, approximately a factor of 2 smaller than in transmission. This is expected given the longer length the light travels inside the scattering medium, and hence the faster deterioration of the signal-to-background ratio. Regarding contrast, ultrasound enables to maintain a value close to 1, up to an optical thickness of 5. At this point, the contrast using the external focusing element is about 0. The more pronounced benefit of ultrasound in reflection mode can be explained by the light-guiding effects on both incident and back-scattered light. Note that maximizing contrast plays a key role in reducing photodamage in applications involving light-sensitive media such as biological tissue.

### Laser scanning microscopy with ultrasound focusing

**Figure 4:**
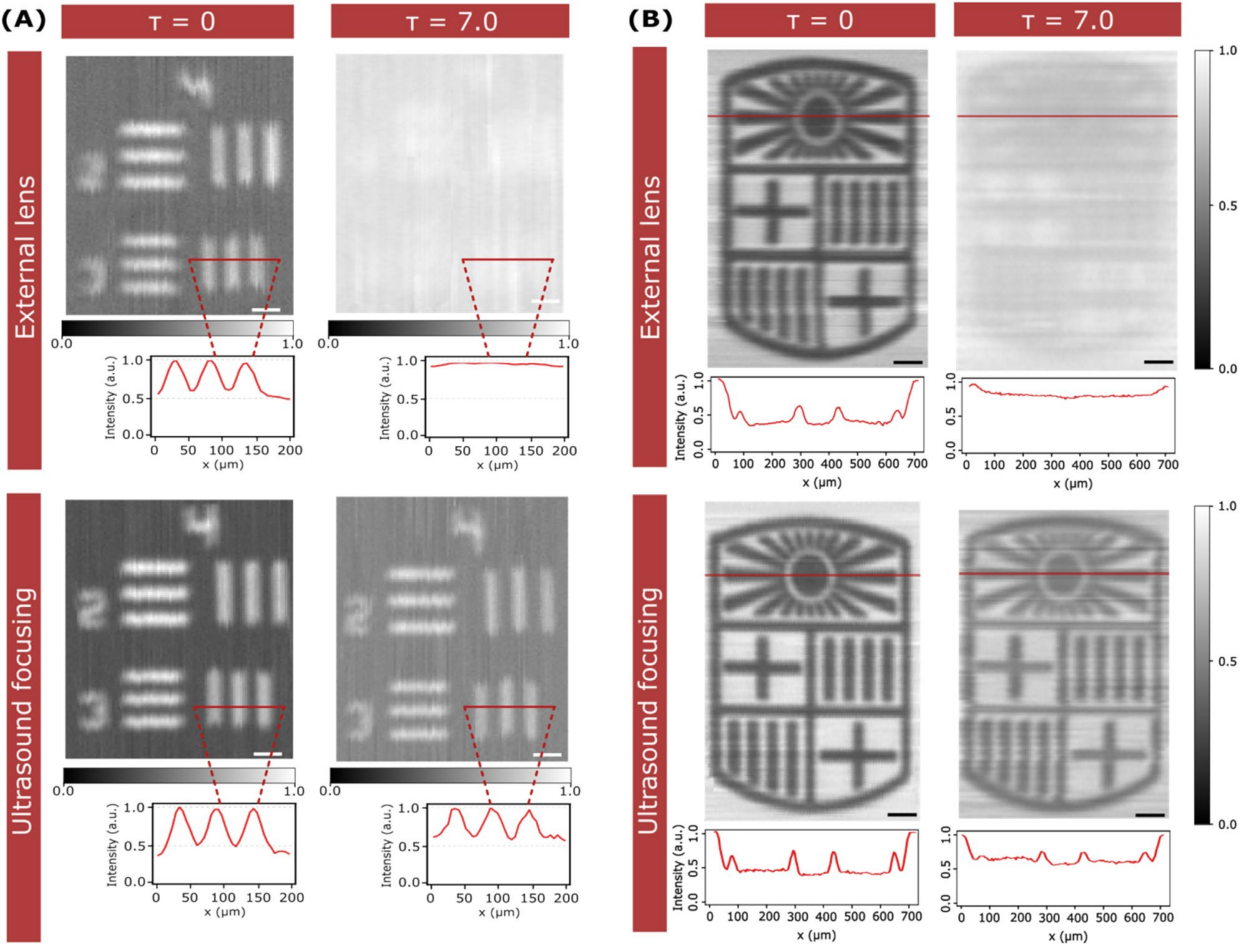
Imaging with ultrasound-focused light. (**A**) Reconstructed images of a USAF target group 4 elements 2 and 3 acquired by scanning the sample, point-by-point, across the focus obtained with an external lens (top) and ultrasound focusing (bottom) inside a homogeneous medium (left) and a turbid medium with τ = 7.0 (right). The insets correspond to the intensity profiles of the selected regions. Scale bars are 50 μm. (**B**) Reconstructed images of the UB (Universitat de Barcelona) logo acquired under the same conditions as (**A**). The insets correspond to the intensity profile highlighted with a line. All images are obtained in reflection mode. Scale bars are 100 μm.

The ability to confine light into a small spot is a requirement for many imaging applications. In particular, for those based on raster scanning a laser across the sample. To analyze how ultrasound can help to improve image formation in the presence of scattering, we captured a series of brightfield images in reflection mode by point-by-point scanning the focused beam across the sample (see Materials and Methods). In all cases, we used as our sample a test target (1951 USAF test chart, see Supplementary Fig. S9) immersed inside water and in a water/milk mixture with τ = 7.0. Different reconstructed images of the test chart using ultrasound and an external focusing element are shown in Fig. 4A. As expected, in a homogeneous medium, no significant differences are observed between images acquired using the two focusing methods. In both cases, the bars that feature a width of 27.84 µm and 24.80 µm (group 4, elements 3 and 4) can be discerned, in agreement with the spatial resolution of our system (25 µm). Instead, the quality of the reconstructed images inside the scattering medium is stunningly different for the two methods used. When imaging using an external focusing element, the contrast is too low to discern any recognizable feature. With ultrasound focusing, the object bars can be clearly distinguished, even the closest ones. In fact, except for a slight loss of contrast, there are no significant differences between images captured with the object immersed in the scattering versus the non-scattering solution. Remarkably, the benefits of ultrasound focusing inside scattering media are still present for imaging narrower bars, down to a width of 15.63 µm (USAF group 5, element 1), as shown in Supplementary Fig. S7.

An additional example of the possibilities of using ultrasound focusing for imaging arbitrary samples immersed in a turbid media is shown in Fig. 4B. In this case, we reconstructed images of a more challenging sample, namely, a metallic logo of our university (700 µm × 1070 µm), see Supplementary Fig. S9. Again, we compared ultrasound with traditional light focusing in reflection mode (additional images obtained in transmission mode are shown in Supplementary Fig. S8). With the sample immersed in a homogeneous medium, both methods can resolve the logo, even its finest structures. In contrast, in the presence of scattering, the performance of ultrasound focusing is plainly superior. Not only is impossible to extract any useful information from the image captured with the external lens, but the image with ultrasound focusing has almost the same resolution as when working in a homogeneous medium. These results further confirm the use of ultrasound focusing as a promising technology for deep light imaging.

## Discussion

Ultrasound inside a scattering medium can be used to locally modulate the optical properties of the medium, effectively acting as an embedded lens or waveguide to focus light. As our results demonstrate, such modulation enables light focusing down to a 25 µm spot inside a turbid medium with an optical thickness up to 15.3. This value corresponds to a depth of around 15 MFP, that is, close to the point where photons reach a diffusive regime. Instead, when using the classical method for light focusing based on a lens placed outside the scattering medium, the spatial resolution reached is up to 7 times worse, with an abrupt decrease in contrast. These results are in agreement with Monte Carlo simulations, further validating the role of ultrasound as a method for redirecting scattered photons to an area of interest. When combined with a scanning system, ultrasound focusing enables imaging samples immersed in turbid media that would be completely hidden with conventional imaging methods. The approach can be implemented in both transmission and reflection configurations.

Compared to existing methods for deep light focusing inside inhomogeneous media, ultrasound offers a high temporal resolution, only limited by the speed of sound in the medium of interest – typically microsecond response times are possible. In addition, ultrasound can be regarded as a safe and non-invasive technology. In current experiments, the pressures used were around 1 MPa (see Supplementary Information and Fig. S10), well-below the safety limit defined by the Food and Drug Administration (FDA) that stipulates rarefaction pressures lower than 5.4 MPa^31^. Importantly, by using higher ultrasound pressures, but still below the FDA limit, higher light confinement is expected. Therefore, we expect our method to be suitable for in vivo characterization and modification of biological samples. We anticipate that ultrasound focusing will lead to an unprecedented control of light inside scattering media. Combined with state-of-the-art techniques for deep light imaging and laser processing, it will allow the real-time study and modification of heterogeneous systems at the microscale, helping to expand the portfolio of applications of light-based methods.

## Materials and methods

### Ultrasound focusing setup

The main components of the setup are depicted in Fig. 1B. A diode laser with a wavelength of 660 nm (Coherent Obis) was used as the light source. The laser enables amplitude modulation up to 150 MHz, effectively achieving laser pulses with a duration of around 2 ns. The laser beam was reduced with a 4f. system of 0.35X magnification and then guided to a cylindrical cavity with an inner diameter of 16 mm and a length of 20 mm made of the piezoelectric material PZT (lead zirconate titanate). The sample was placed at the output of the cavity, using a sample holder attached to an XYZ stage with a precision of 0.2 µm (Physik Instrumente, Apollo). The transmitted beam was directed toward a charged-coupled device (CCD) camera (Thorlabs, DCU224M) via a microscope with 2X magnification. The reflected beam was redirected via a beam splitter toward a microscope with 1.6X magnification and a second CCD camera. To generate the ultrasound waves inside the cavity, the piezoelectric was driven on resonance using an arbitrary waveform generator (AWG) (SIGLENT SDG6022X). The synchronization signal of the AWG was used to trigger the laser pulses. An in-house Labview program was used to control the position of the stage and synchronize it with camera acquisition.

### Image formation of the samples

All images were acquired by translating the sample relative to the ultrasound cavity in a point-by-point fashion. The distance between adjacent points was in all cases 10 µm, which determined the sampling of the reconstructed image. At each position, the reflected or transmitted light from the sample was captured with the corresponding CCD camera. For speeding up image acquisition, only a region of interest of 30 × 30 pixels was acquired, as shown in Supplementary Fig. S8. The images are finally reconstructed by assigning to each position the average CCD signal. The laser power for each imaging process ranges from 2.8 µW to 1.35 mW and the exposure time varies between 0.01 ms to 5 ms. Line artifacts caused by line-to-line intensity bias were partially removed by subtraction. All data post-processing was performed in Python.

### Tissue phantom preparation and characterization

The tissue phantoms were prepared by diluting fat milk in pure water. The water/milk mixture enables easy tuning of the scattering properties by simply changing the concentration of milk in the solution. The highest mixture of milk was obtained at a concentration of 2:100. The scattering properties of the tissue phantoms were determined by analysis of the light attenuation as a function of thickness^32^, from which the scattering coefficient and optical thickness of the mixture are obtained (see “Supplementary Information”).

### Beam propagation simulations

The focusing of light inside a homogeneous medium was simulated using the beam propagation method (BPM). This algorithm is used to estimate the propagation of a light beam in a medium with small variations in refractive index. The simulation parameters for BPM with ultrasound focusing were: 660 nm wavelength, incident Gaussian beam with 300 µm waist, propagation distance of 2 cm, and refractive index amplitude (see Eq. 1) of ${n}_{A}$ =8·10^–5^ with a frequency of 4 MHz and a static refractive index of 1.33 (water). In the case of an external focusing element, light was initially propagated through the ultrasound cavity with the same parameters as before but with ${n}_{A}$=2·10^–5^. Then, it was further propagated with no ultrasound for another 2 cm. The BPM algorithm was implemented in a Matlab environment.

### Monte Carlo simulations

The propagation of light inside a scattering medium was simulated using a recently published Monte Carlo implementation^26^. The algorithm is based on the photon packet method. The properties of the medium are locally specified on a triangular grid geometry, namely the scattering coefficient (µ_s_), absorption coefficient (µ_a_), the scattering anisotropy factor (g), and refractive index (n). In this work, different scattering coefficients ranging from 0.5 to 5 cm^−1^ were used. In all cases, absorption was considered negligible^7^ and the anisotropy factor value selected was g = 0.94^7^. The photon source had a length of 300 µm and a small Gaussian divergence of σ = 6·10^–3^ mm. For ultrasound focusing, the refractive index distribution was that reported in Eq. 1, with ${n}_{A}$=7·10^–5^ at a frequency of 4 MHz. The static refractive index of water was considered. In this case, the simulated region was 2 cm long and 500 µm wide, with a grid size of 4 µm. For simulating the focusing with an external lens, the selected length of the simulation region was changed to 4 cm, with a refractive index modulation of ${n}_{A}$=1·10^–5^ during the first 2 cm of photon transport, and no modulation during the remaining 2 cm. The Monte Carlo algorithm was implemented in Matlab.

### Modulation transfer function (MTF) determination

The MTF was obtained by the slanted-edge method. In this approach, three main steps are considered (see Supplementary Fig. S6). First, a knife-edge target is imaged, which reveals the system response to a sharp edge—the edge spread function (ESF). Secondly, the line spread function (LSF) is obtained from the numerical derivative of the ESF. Finally, the MTF is calculated from the Fourier Transform of the ESF^29^. The cut-off frequency of the system was estimated using the criterion MTF = 0.1. The processing of the MTF was performed in Matlab.

## Supplementary Information

Supplementary Information.

## Data Availability

The datasets used and/or analyzed during the current study available from the corresponding author on reasonable request.
