# Supplementary material for: Enhanced light focusing inside scattering media with shaped ultrasound
Source: Sci Rep. 2023 Jul 17;13:11511. doi: 10.1038/s41598-023-38598-5 (PMC10352373; doi:10.1038/s41598-023-38598-5)
Supplement: Supplementary file 1 — Supplementary Information. [file 41598_2023_38598_MOESM1_ESM.docx]

**Supplementary information:**

**Enhanced light focusing inside scattering media with shaped ultrasound**

Blanca Mestre-Torà^1^, Martí Duocastella^1,2^*

- 1. Department of Applied Physics, Universitat de Barcelona, C/ Martí i Franquès 1, 08028 Barcelona, Spain.

1. Institut de Nanociència i Nanotecnologia (In^2^UB), Universitat de Barcelona, 08028 Barcelona, Spain.

* Corresponding author. Email: [marti.duocastella@ub.edu](mailto:marti.duocastella@ub.edu) (M.D.)

1. Experimental measurement of the scattering properties of the water/milk mixtures

To measure the scattering coefficient ($\mu_{s}$) of the water/milk mixtures, we measured the light attenuation as a function of the medium thickness. Specifically, we placed a mirror inside a reservoir filled with the turbid medium and collected the reflected light intensity at different mirror-reservoir distances, as shown in Figure S2A. Considering single-scattering regime, the observed light attenuation as light travels a longer distance inside the medium is expected to follow an exponential decay given by the Beer-Lambert law (1). At conditions where light attenuation is negligible - as in current experiments, where absorption is a factor of 100 smaller than scattering (2) - the attenuation coefficient is solely due to scattering. Thus, from the plot of the collected light versus the medium thickness, it is possible to determine the scattering coefficient $\mu_{s}$ with a single exponential fitting (Figure S2B). Importantly, to ensure single-scattering regime, the attenuation factor is measured within the region where the light intensity is attenuated from 1 to 0.6, where we can ensure scattered photons underwent only one scattering event. Once $\mu_{s}$ is determined, the optical thickness ($\tau$) of the medium is calculated as$\tau=\mu_{s}d$, where d corresponds to the medium thickness the light traverses.

2. Contrast and contrast loss parameters for Monte Carlo simulations

We defined two metrics to evaluate the light focusing effects on the Monte Carlo simulations. The first one, named focusing contrast ($f_{contrast}$), was defined as the difference between the number of focused photons (given by the fluence at the focus, $\varphi_{focus}$) and the number of background photons (given by the fluence at the vicinity of the focus, $\varphi_{background}$) relative to the overall number of photons ($\varphi_{focus}$+$\varphi_{background}$).

$f_{contrast}=\frac{\varphi_{focus}-\varphi_{background}}{\varphi_{focus}+\varphi_{background}}$ (1)

The second metric was used to quantify the deterioration of the system focusing capability inside the scattering media. Named contrast loss ($\Delta f_{contrast}$), it consisted of the relative difference in focusing contrast between a homogeneous ($f_{cH}$) and a scattering ($f_{cS}$) medium.

$\Delta f_{contrast}=\frac{f_{cH}-f_{cS}}{f_{cH}}$ (2)

3. Refractive index modulation and pressure variation relationship

The local variation of pressure in water produces a local variation of the refractive index. The relationship between these parameters can be explicitly written as (3):

$n\left( \tilde{p} \right)=\sqrt{\frac{1+2\left( a_{9}+({a_{9}+a}_{10})\tilde{p}+({a_{7}+a}_{10})\tilde{p}^{2}+a_{7}\tilde{p}^{3} \right)}{1-\left( a_{9}+({a_{9}+a}_{10})\tilde{p}+({a_{7}+a}_{10})\tilde{p}^{2}+a_{7}\tilde{p}^{3} \right)}}$ (3)

where $n$ corresponds to the refractive index, $a_{i}$ are the dimensionless coefficients given in (4), and $\tilde{p}$ is defined as a dimensionless pressure produced by the acoustic fluctuations. Thus, an estimation of the pressure variation in water can be obtained knowing the induced changes in refractive index, as shown in Fig. S10. Considering a variation in the refractive index of $\Delta n=8\cdot{10}^{-5}$ (see Results section in the main text), the maximum effective variation is $\Delta n=16\cdot{10}^{-5}$ which yields a maximum pressure change of $\Delta p=1.1 MPa$ in the medium.


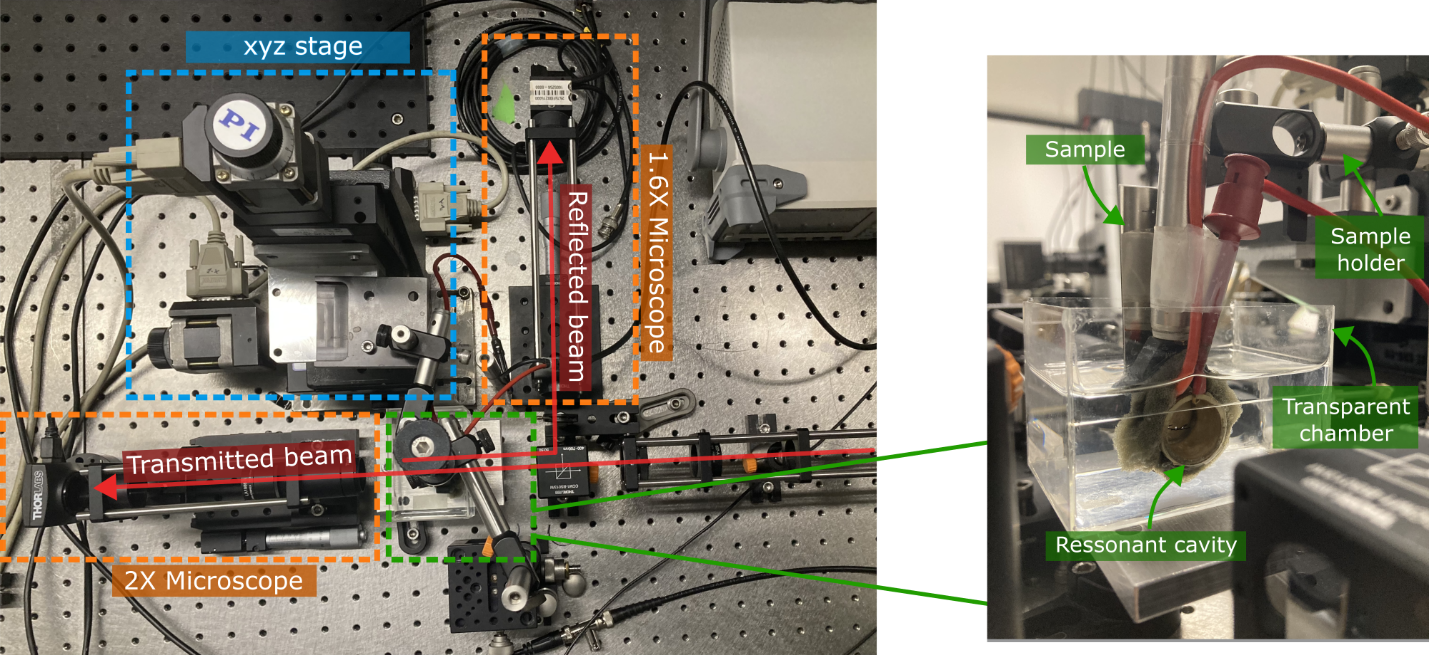


Fig. S1.

**Photograph of the experimental set-up used in this work.** The different parts can be distinguished as follows: (**--**) xyz stage, (**--**) microscopes, (**--**) focusing structure, and (--) light beam. The inset on the right corresponds to a close-up photo of the piezoelectric resonant cavity with its holder, the transparent and liquid filled chamber where it was immersed, and the sample and corresponding holder.


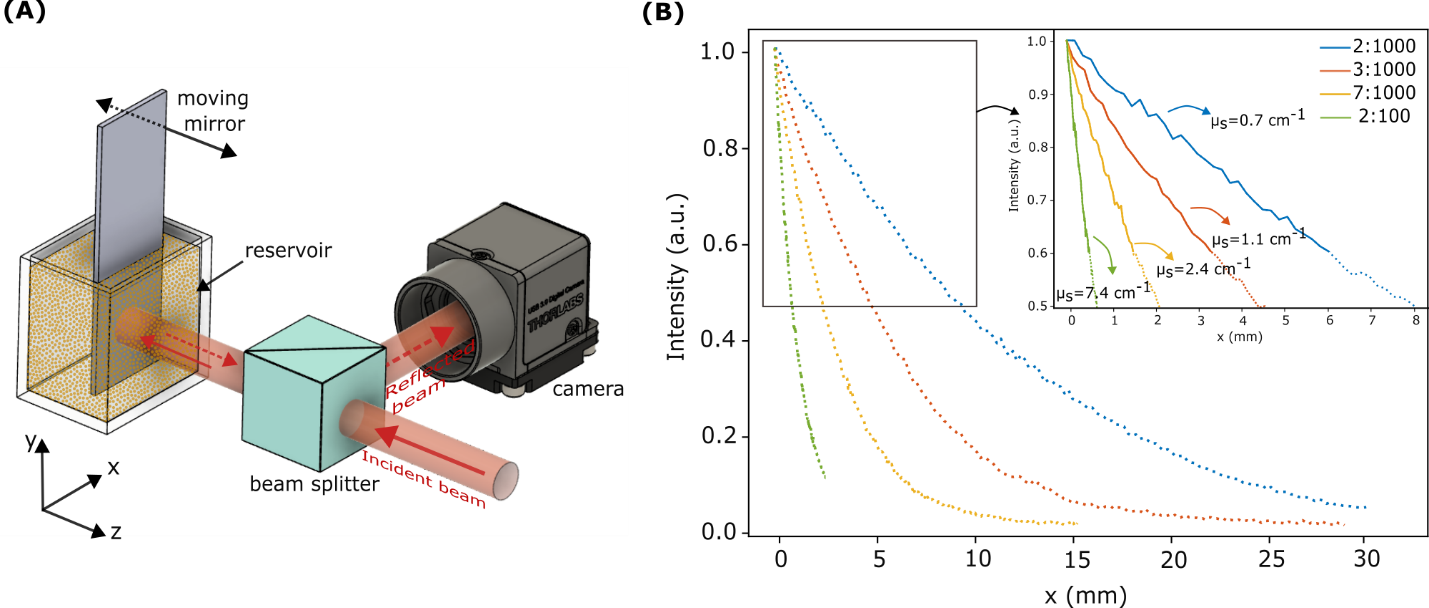
Fig. S2.

**Experimental measurement of the scattering coefficient of the water/milk mixtures.** **(A)** Scheme of the setup used to perform the measurement. The mirror was moved using a motorized translation stage (not shown). **(B)** Plot of the light intensity versus the thickness of water/milk mixtures. The inset also shows the computed scattering coefficient of each mixture. The mixtures correspond to milk/water dilutions of (--) 2:1000, (--) 3:1000, (--) 7:1000, and (--) 2:100.


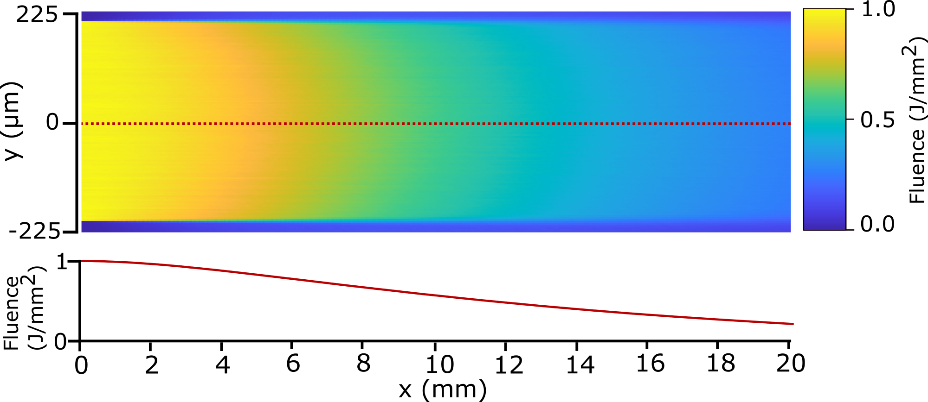


**Fig. S3.**

**Monte Carlo simulations of the beam propagation in a medium with an optical thickness of 2.** Colormap plot and fluence profile of the simulated results obtained when the beam propagates in the scattering medium without ultrasound. The simulated area was 2 cm long and 500 µm wide, with a grid size of 4 µm.

**
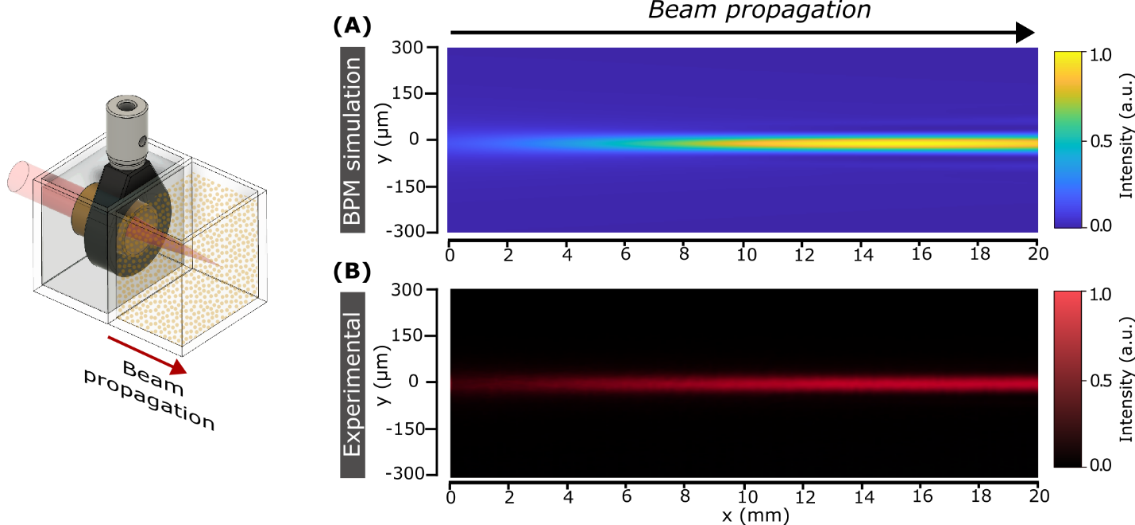
**

**Fig. S4.**

**Propagation of a Bessel-like beam inside a homogeneous medium generated using an external focusing element. (A)** Intensity colormap of the simulation using the Beam Propagation Method (BPM). The external focusing lens is a water-filled ultrasound cavity with a 16 mm diameter, driven at a resonant frequency of 4 MHz, and producing a change in refractive index of $\Delta n={2\cdot10}^{-5}$(see Equation 1, main text). The laser source simulated has a Gaussian profile with a beam width of 300 µm and a wavelength of 660 nm. **(B)** Experimental measurement of the light intensity at the conditions simulated in (A). Note the very good agreement between experiment and simulation.


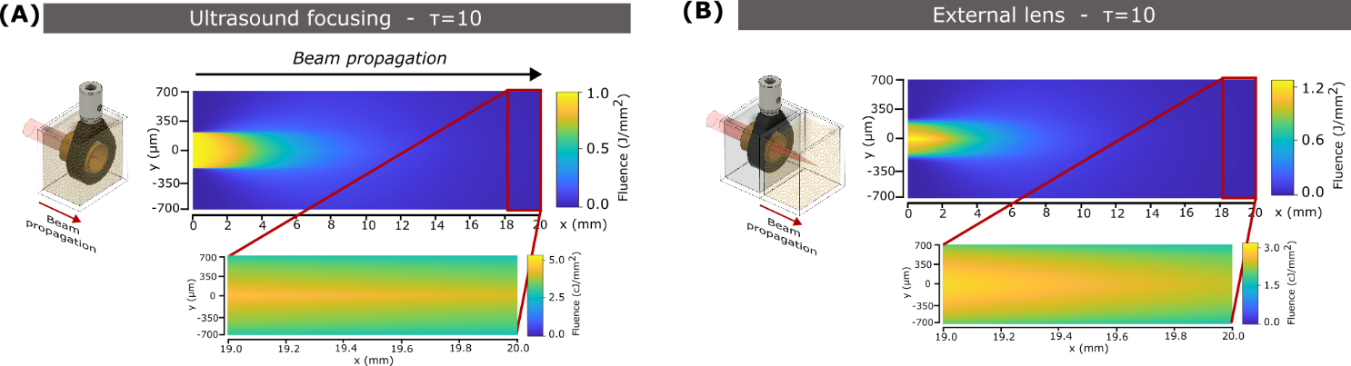
 **Fig. S5.**

**Monte Carlo simulations of the beam propagation in a medium with an optical thickness of 10.** **(A)** Colormap plot of the simulated results obtained when using ultrasound focusing. The medium was modulated with a Bessel refractive index profile (see Equation 1, main text) with $\Delta n=8\cdot{10}^{-5}$, $n_{0}=1.33$, a frequency of 4 MHz, µ_s_ = 5 cm^-1^, an anisotropy factor of 0.94 and no absorption. The simulated area was 2 cm long (hence, the optical thickness of the medium is 10) and 1.5 mm wide, with a grid size of 0.01 mm. **(B)** Colormap plot of the simulated results with an external focusing element. In this case, the simulated area was 4 cm long and 1.5 mm wide, with a grid size of 0.01 mm. During the first 2 cm, a Bessel refractive index profile as in (A) was considered, but with $\Delta n=1\cdot{10}^{-5}$ and a constant refractive index of $n_{0}=1.33$. The last 2 cm of the simulated area were considered to have µ_s_ = 5 cm^-1^, so τ=10. Note that, when using an external focusing element, light confinement clearly deteriorates after 2 cm propagation in a scattering medium compared to ultrasound focusing.


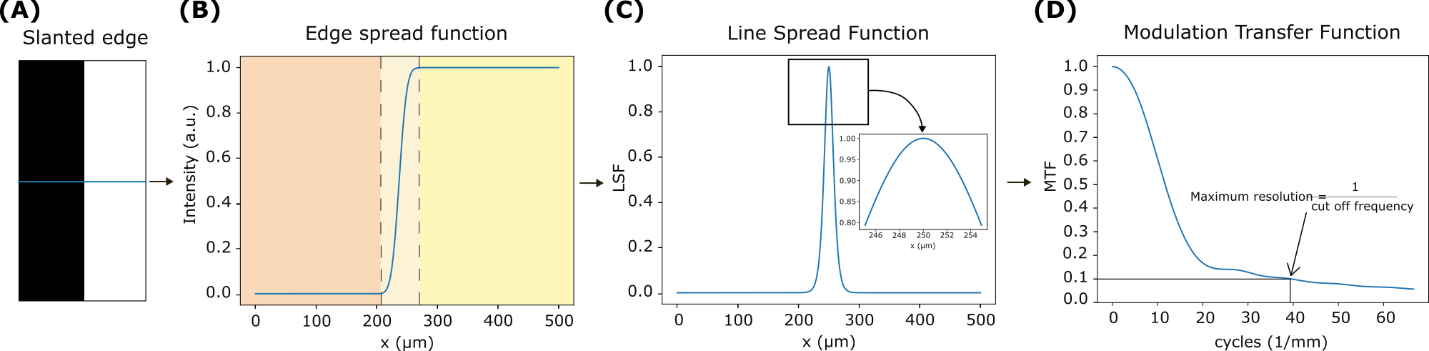
**Fig. S6.**

**Scheme of the process to obtain the modulation transfer function (MTF) of the optical system via the slanted-edge method.** **(A)** Image of a knife-edge target, from which the MTF is processed following three steps. **(B)** Line profile of the knife-edge region target, it corresponds to the edge spread function (ESF). **(C)** The line spread function (LSF) of the system, which is obtained from the derivative of the ESF. **(D)** MTF of the system, it is calculated via the Fourier Transform of the LSF (5,6). The MTF decreases when increasing the spatial frequency, down to a value where objects are not discernible - the so-called cutoff frequency, which determines the maximum spatial frequency the optical system can resolve, namely, the resolution of the system. The cut-off frequency is defined at an MTF value of 0.1 (6). From the cut-off frequency, the maximum resolution of the system is obtained as it corresponds to the inverse value of the latter.


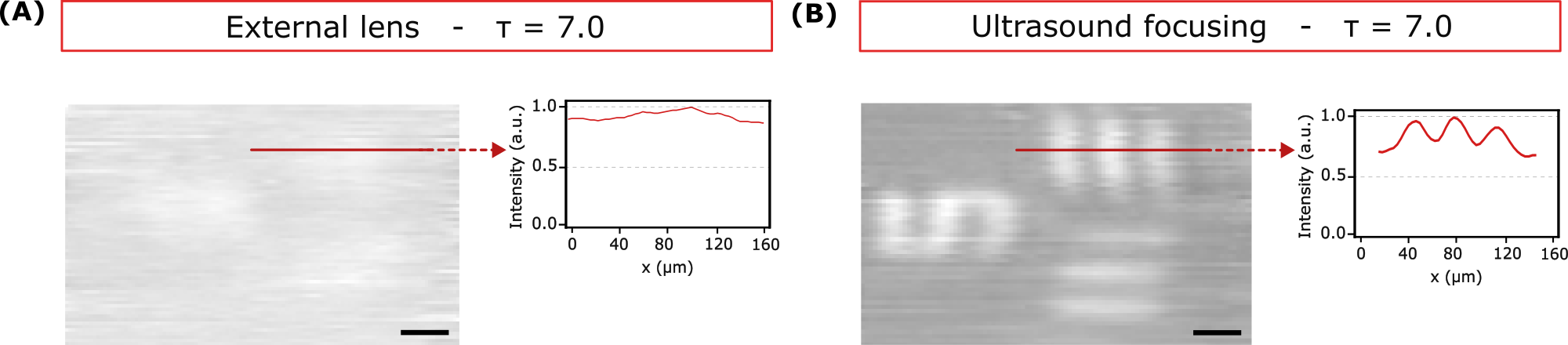


Fig. S7.

**Images in reflection mode. (A)** Optical micrograph of the USAF target group 5 element 1 obtained using an external focusing element and the sample immersed in a medium with an optical thickness of 7. **(B)** Optical micrograph of the same region as in (A) but using ultrasound focusing. The imaged region has a bar width of 15.63 μm, approximately half of the spatial resolution – around 25 μm - of the focusing system.


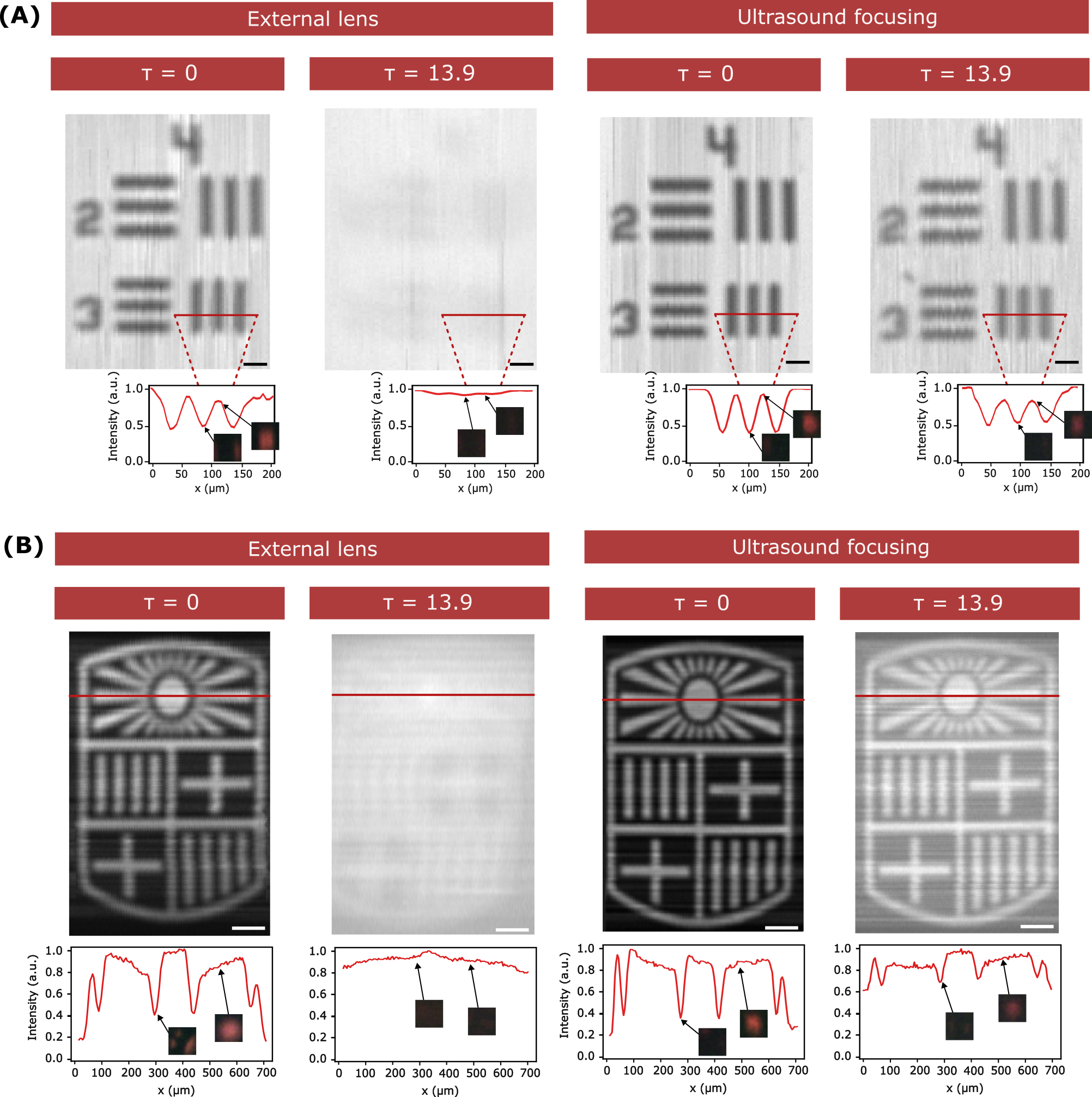


Fig. S8.

**Imaging in transmission mode**. **(A)** Optical micrographs of a USAF target group 4 elements 2 and 3 obtained in transmission mode using an external lens (left) and ultrasound focusing (right) when the sample is immersed in a 2 cm thick homogeneous medium (τ=0) and a 2 cm thick water/milk mixture with τ=13.9. The insets correspond to the intensity profiles of the selected regions, where it is also shown the CCD signal obtained for each position of the image. Scale bars are 50 μm. **(B)**. Optical micrographs of the University of Barcelona logo obtained in the same multiple conditions as in (A). Scale bars are 100 μm.
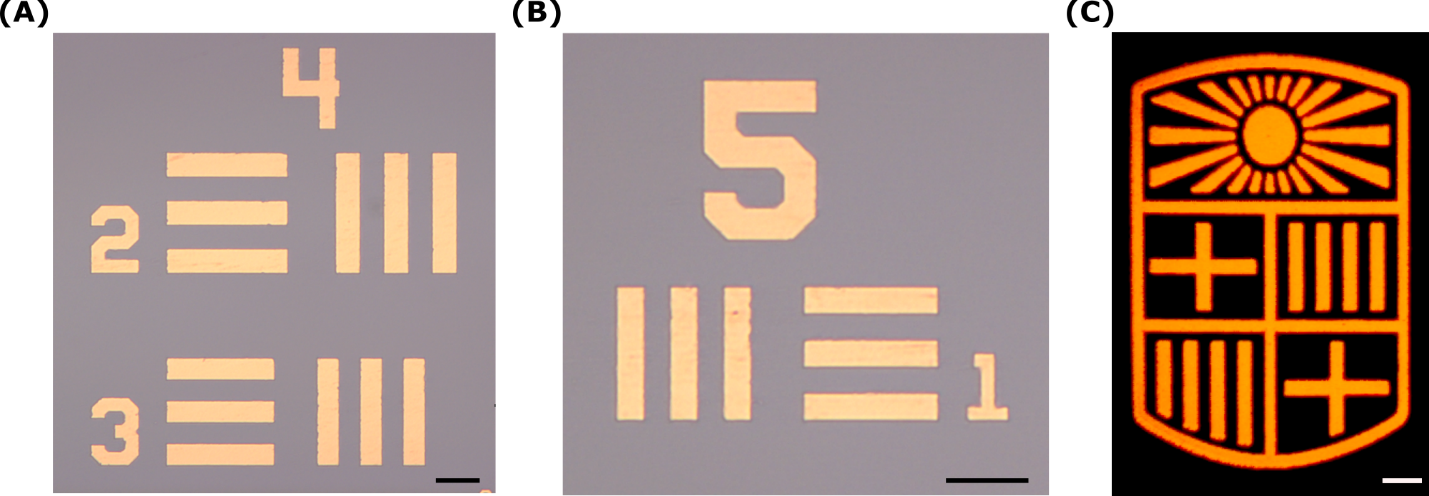
**Fig. S9.**

**Brightfield microscope images of the samples used in this work**. **(A)** Optical micrograph of the USAF group 4 elements 2 and 3. **(B)** Optical micrograph of the USAF group 5 element 1. **(C)** Optical micrograph of the University of Barcelona logo. Scale bars are 50 μm, 50 μm and 100 μm respectively.


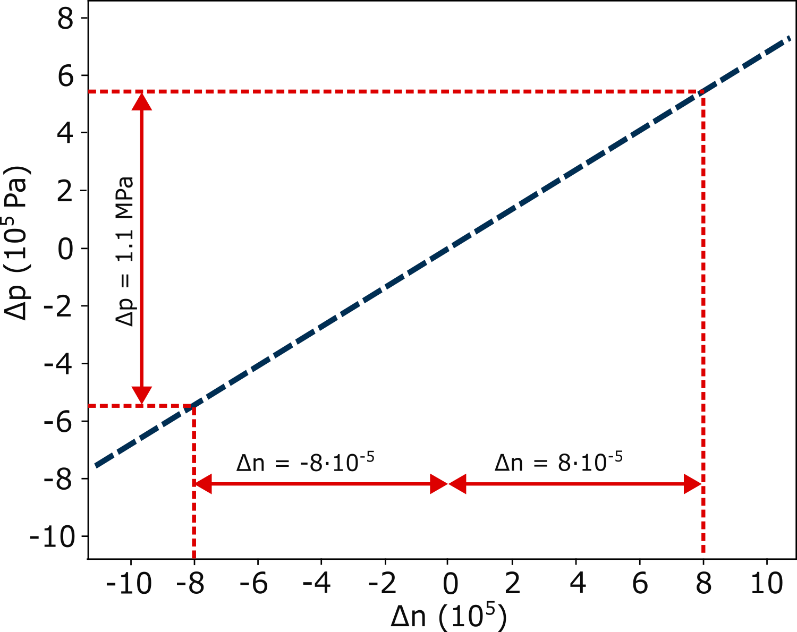


**Fig. S10**.

**Relationship between the refractive index variation and pressure changes in water.** Plot of the variations in pressure versus the change in refractive index. At the conditions considered herein, there is a linear relationship between the two.

References

​​1. M. Bhatt, K. R. Ayyalasomayajula, P. K. Yalavarthy. Generalized Beer–Lambert model for near-infrared light propagation in thick biological tissues. *J Biomed Opt* **21**, 076012 (2016).

​2. B. Aernouts, R. Van Beers, R. Watté, T. Huybrechts, J. Lammertyn, W. Saeys. Visible and near-infrared bulk optical properties of raw milk. *J Dairy Sci* **98**, 6727–6738 (2015).

​3. J. N. Caron, G. P. DiComo. Frequency response of optical beam deflection by ultrasound in water. *Appl Opt* **53**, 7677 (2014).

​4. International Association for the Properties of Water and Steam (IAPWS) (1997).

​5. X. Xie, H. Fan, A. Wang,  N. Zou, Y. Zhang. Regularized slanted-edge method for measuring the modulation transfer function of imaging systems. *Appl Opt* **57**, 6552 (2018).

​6. G. Bostan, P.E. Sterian, T. Necsoiu, A. P. Bobei, C. D. Sarafoleanu. The slanted-edge method application in testing the optical resolution of a vision system. *Journal of optoelectronics and advanced materials* **21**, 22-34 (2019).
